# Selection and optimization strategy for Rap1-targeting single-domain antibodies as platelet activation markers

**DOI:** 10.1016/j.rpth.2025.103294

**Published:** 2025-12-11

**Authors:** Marie-Christine Alessi, Maxime Moulard, Daniele Boulay-Moine, Cyril Pons, Marielle Margier, Cléa Vessière, Marjorie Poggi, Theo Pigaglio, Francoise Dignat-George, Alain Roussel, Stéphane Burtey, Laurent Bonello, Patrick Chames, Remi Bonjean, Franck Peiretti

**Affiliations:** 1 Aix Marseille University, C2VN, INSERM, INRAE, Marseille, France; 2 BioCytex, Marseille, France; 3 Aix Marseille University, CRCM, CNRS, INSERM, Marseille, France; 4 Aix Marseille University, LISM, CNRS, Marseille, France

**Keywords:** antibodies, GTP, human, platelet activation, Rap1

## Abstract

**Background:**

Rap1 is critical for platelet activation, functioning as a key node of the platelet activation pathways.

**Objectives:**

This study aimed to develop VHH-Fc (minibodies) against Rap1 for the purpose to quantify active Rap1 levels in platelets.

**Methods:**

We have produced the first generation of VHHs against active Rap1 through a series of negative and positive screenings of a synthetic phage display library, utilizing both inactive and active Rap1B. We performed random mutagenesis, followed by yeast 2-hybrid screening to optimize variants.

**Results:**

Among 122 VHH clones, 2 with the highest redundancy were subcloned as VHH-Fc. Both selectively detected active Rap1B G12V in HeLa cells but failed to recognize the inactive Rap1B S17N isoform. They successfully captured Rap1 from platelet lysates incubated with GTPγS and from thrombin receptor activator peptide 6–stimulated platelets. Further optimization yielded 2 superbinder VHH clones: VHH-Fc B89 and VHH-Fc B14. VHH-Fc B89 exhibited a *K*_D_ of 4.4 nM and showed enhanced capacity to capture active GTP-bound Rap1 compared with the original VHH-Fc. Additionally, it was able to detect overexpressed active Rap1B G12V in HeLa cells. An ELISA setup combining VHH-Fc B14 and a commercial monoclonal antibody targeting total Rap1 was highly effective in detecting both GTPγS-bound Rap1 and endogenous active Rap1 in platelets.

**Conclusion:**

This study identifies, that accurately capture active Rap1 in platelets, thereby establishing them as promising tools for future research. We also developed a reliable ELISA test that can facilitate clinical studies to monitor platelet Rap1 activation in various medical contexts.

## Essentials

• Rap1 is a critical GTPase involved in platelet activation.
• A synthetic phage display library was screened to identify VHH targeting active Rap1.
• Several VHH-Fc were found to effectively capture active Rap1 in platelets.
• A VHH-fc–based ELISA assay was developed than accurately quantify endogenous active Rap1 in platelets.

## Graphical abstract

**Figure:**
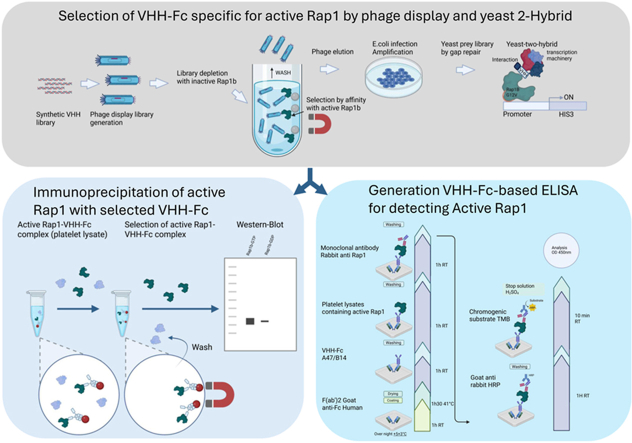

## 1 Introduction

Platelets play a crucial role in primary hemostasis and thrombus formation in various pathologic conditions such as acute coronary syndrome and stroke [1]. Elevated expression levels of activation-dependent platelet markers have been used to assess the risk for developing thrombosis. The primary methods employed for this evaluation involve measuring receptors on the platelet surface, such as CD62P (P-selectin) and soluble platelet markers in plasma, including soluble P-selectin and soluble CD40L. Additionally, circulating platelet-leukocyte aggregates and platelet microvesicles have been quantified in different clinical settings [2]. Each of these methods has its advantages and limitations—some lack sensitivity, while others focus on only 1 aspect of platelet function. The development of new, more integrative markers is likely to provide additional evidence of platelet activation status and may further elucidate the underlying causes of thrombogenic events in certain diseases.

The Rap protein family consists of the small GTPases Rap1A, Rap1B, Rap2A, Rap2B, and Rap2C [3]. These proteins are involved in various physiological processes such as immune responses, hematopoiesis, neuronal development and plasticity [4,5], tumorigenesis [4,6,7], and platelet function.

Rap1 isoforms are the most abundant GTPase found in platelets, with 125,000 copies of Rap1A and 154,000 copies of Rap1B per platelet, whereas all Rap2 isoforms combined account for a total of 12,000 copies per platelet [8]. Rap1 is critical for platelet activation, downstream of calcium release and part of the common platelet activation pathway. Combined deficiency of RAP1A and RAP1B markedly impairs platelet aggregation, spreading, and clot retraction, whereas this is not the case in deficiency of only 1 RAP1 isoform [9]. Both Rap1 isoforms significantly contribute to thromboxane A2 generation and the inside-out activation of platelet integrins [10,11]. Therefore, quantifying the active pool of Rap1 could be a clinically significant biomarker to monitor platelet activation, which may provide a more comprehensive evaluation of overall platelet activation levels than currently available markers.

Rap1 cycles between an inactive state bound to GDP and an active state bound to GTP. Thus, assessing Rap1 activation involves quantifying the GTP-bound form or its capacity to bind GTP, even though the proper activity of these small GTPases is the hydrolysis of GTP [12]. The paucity of studies formally reporting the active fraction of Rap1 in patient cohorts is likely due to the limitations of the standard tools available to evaluate GTP-bound Rap1 levels. Upon GTP binding the conformation of GTPase is altered such that it can associate with downstream effectors molecules. Several of these effector proteins contain Ras binding domains that have a 100-fold preference for the GTP over GDP. Based on these findings, the current main method for estimating the amount of active Rap1 involves capturing the GTP-bound Rap1 using the Rap1 binding domain of RalGDS, a guanine nucleotide exchange factor, in pull-down experiments, followed by western blot analysis [13]. However, the pull-down assay is a complex technique that requires considerable time and resources to obtain reliable results, limiting its use to specialists for research and preventing the widespread use of active Rap1 determination on a large scale such as in clinical studies.

In this study, we isolated and characterized high-affinity single-domain antibodies, also called VHH or nanobodies, which bind specifically to the GTP-bound fraction of Rap1 by panning the NaLi-H1 phage display library displaying humanized synthetic single-domain antibodies [14] against a constitutively active mutant of Rap1B and against GTP loaded Rap1B. We developed a conjugate comprised of VHH targeting Rap1 fused to an Fc domain (VHH-Fc [minibodies]). The VHH-Fc fusion proteins were able to selectively detect GTP-bound Rap1 in an immunoprecipitation assay using platelet extracts. A VHH-Fc–based capture ELISA assay was developed and used to measure GTP-bound Rap1 levels in platelets.

## 2 Methods

### 2.1 Rap1 B antigen purification

The coding sequence of the human wild-type Rap1B (Rap1B WT; NM_001010942.3) and those of the constitutively inactive (Rap1B S17N) and active Rap1B (RAP1B G12V) mutant forms of Rap1B with the C181A mutation in the CAAX C-terminal motif were cloned into the *Escherichia coli* expression vector pEB7 (pMAL-C2; New England Biolabs) downstream and in frame of the sequence coding for the maltose binding protein (MBP)-HA double tag (Supplementary Figure 1A). BL21DE3 *E coli* transformed with pEB7 plasmids were cultured in LB medium until the optical density at 600 nm reached 0.8 to 1, then induced with 0.5 mM Isopropyl β-D-1-thiogalactopyranoside, and grown overnight at 16 °C. Bacteria were harvested by centrifugation at 4000*g* for 20 minutes, suspended in phosphate-buffered saline (PBS), pH 7.4, supplemented with 10% glycerol and 1% Triton X-100, and lysed by sonication on ice prior to centrifugation (30 minutes, 15,000*g*, 4 °C). Recombinant proteins were purified using affinity chromatography on amylose resin (New England Biolabs) in batch mode according to the manufacturer’s instructions. The elution fractions containing the recombinant fusion protein were pooled and dialyzed overnight at 4 °C against PBS, pH 7.4.

The homogeneity of the samples was checked by Coomassie blue staining of SDS-PAGE gels, and the image of the gel were captured by a gel imager FluorSMax (Bio-Rad) (Supplementary Figure 1B). Both purified MBP-HA Rap1B WT loaded with GTPgS and MBP-HA Rap1B G12V were able to bind RalGDS-RBD, whereas MBP-HA Rap1B S17N was not (Supplementary Figure 1C).

### 2.2 VHH selection and optimization

The synthetic phage display NaLi-H1 library was used to select VHH against active Rap1B. The VHH A47 was matured by random polymerase chain reaction (PCR) mutagenesis, followed by yeast 2-hybrid screening to identify VHHs with improved binding properties.

### 2.3 Generation of bivalent VHH-Fc

VHH-Fc were produced as fusion proteins with the Fc domain of murine or rabbit IgG2b [15] and with the Fc domain of human IgG1 silenced for FcγR binding (Fc Silent [FcS]) [16]. Briefly, the VHH coding sequences of candidate clones from the yeast screens were subcloned into pFuse-mouseIgG-Fc2, pFuse-rabbitIgG-Fc2, and pHLSec vectors and transfected in HEK293 cells (X-tremeGENE HP DNA; Merck). HEK293 media containing VHH-Fc was collected after 4 to 5 days of culture without serum. Expected size for VHH-Fc (42 kDa) was verified by western blot using antirabbit Fc and antimouse Fc antibodies.

### 2.4 Immunofluorescence

HeLa cells were grown on poly-L-lysine–coated coverslips in 24-well plates in Eagle's minimal essential medium (Sigma; M2779) containing 10% fetal bovine serum and transfected with GFP-G12V Rap1B plasmid for 48 hours. HeLa cells were fixed in 3% paraformaldehyde and permeabilized with 0.05% saponin and stained with nonpurified VHH-Fc (pFuse vector). Cells were washed quickly twice and incubated with Cy3-labeled secondary antibodies for 30 minutes (Invitrogen–Thermo Fisher Scientific).

### 2.5 Assessment of VHH binding affinity

Biolayer interferometry (BLI) measurements were performed using OCTET R2 (Sartorius). Human Rap1B was first loaded with 10 μM GTPyS (Millipore) or 100 μM GDP (Millipore) along with 2 mM EDTA. The mixture was incubated for 30 minutes at 30 °C with shaking (800 rpm), and the reaction was stopped by adding 10 mM MgCl_2_.

VHH-hFcS fusion proteins were immobilized on a protein A biosensor at 2.5 μg/mL in a buffer containing 20 mM Tris-HCl, pH 7.4, 0.1% (v/v) BSA, 200 mM NaCl, 2 mM EDTA, and 10 mM MgCl_2_ and incubated with increasing concentrations of Rap1B (0.04, 0.13, 0.4, and 1.2 μM). All binding data were collected at 25 °C. The experiments included the following 5 steps: (i) baseline (60 seconds); (ii) VHH-hFcS loading onto sensors (120 seconds); (iii) second baseline (60 seconds); (iv) association of proteins for measurement of *K*_a_ (300 seconds); and (v) dissociation of proteins for the measurement of *K*_d_ (300 seconds). Data were fitted with a 1:1 stoichiometry using Sartorius Octet Analysis studio 12.2 software. As a reference, BLI assays were also carried out by soaking the VHH-hFcS–coated sensor without target protein.

### 2.6 Sample collection and platelet preparation

Blood samples were collected from healthy subjects to conduct an observational study. This study was approved by the southwest ethical committee in June 2020 under the number CPP2020-07-062/2020-A00483-49/20.06.16.56049. The participants were recruited at the Assistance Publique des Hôpitaux de Marseille, who were aged between 18 and 85 years, gave their consent, and were affiliated with the French social security. Blood samples were taken in the morning and obtained in a fasting state. Samples were pseudonymized. Platelet-rich plasma (PRP) was prepared within 3 hours after blood collection. In some cases, PRP was incubated with thrombin receptor activator peptide (TRAP)14 (as indicated in the figures). Washed platelets were prepared as previously described [17]. After centrifugation (170*g*, 10 minutes), the PRP was transferred into tubes containing 30 mL of apyrase (200 IU/mL; Merck) and incubated for 5 minutes at 37 °C. Platelets were pelleted by centrifugation (1000*g*, 5 minutes), resuspended in Tyrode buffer (Sigma; containing 10 IU/mL heparin, 0.02 U/mL apyrase, and 0.5 mM PGE1), and maintained for 1 hour at 37 °C. For each preparation, platelet count was determined using flow cytometry (Accuri; BD). For some experiments washed platelets suspended in Roswell Park Memorial Institute medium (×10^9^ platelets/mL) were stimulated at 37 °C with TRAP6 for 8 minutes at 37 °C. Reactions were stopped with the ice-cold lysis buffer (100 mM Tris HCl pH 7.4, 2% NP-40, 1 M NaCl, 5 mM MgCl_2_, and 10% glycerol, supplemented with a protease inhibitor cocktail; Roche). Platelet lysis was completed on ice for 15 minutes with vigorous shaking. Aliquots of lysates were stored at −80 °C before further processing. For ELISA, PRP samples were diluted in PBS, 0.8%/PEG, 0.035% and Proclin 300 and centrifuged (1200*g*, 10 minutes), washed with PBS, and lysed with lysis buffer before freezing at −80 °C.

### 2.7 Loading of GTPases with GTPγS/GDP

Cell lysates or recombinant GTPases were incubated with 10 mM EDTA and 1 mM GDP or 100 μM GTPγS for 30 minutes at 37 °C or at 30 °C for recombinant proteins. The reaction was stopped by adding 60 mM MgCl_2_, and samples were snap-frozen and stored at −80 °C or used directly for downstream applications.

### 2.8 Pull-down assay

Platelet lysates were centrifuged at high speed, and the supernatants were collected. GTPases activation were determined using commercially Ral-GDS–based pull-down assay kit (Merck), followed by standard western blotting procedure.

### 2.9 Immunoprecipitation assay

Platelet lysates (∼200 μg proteins) were incubated with VHH-Fc–coupled magnetic beads (Pierce Protein A/G; 250 μg/25 μL beads and 1-2 μg VHH-Fc) overnight at 4 °C with gentle agitation. After washing (25 mM Tris HCl 7.4, 150 mM NaCl, 1 mM EDTA, 1% NP 40, 5% glycerol), the beads were mixed with SDS-PAGE sample buffer and heated at 95 °C for 5 minutes. The supernatant was stored at −80 °C until use or subjected to western blotting.

### 2.10 Western blot

Heat-denatured and reduced platelet protein samples were submitted to SDS-PAGE separation on 4% to 12% BisTris NuPAGE gels (Invitrogen) and transferred to polyvinylidene fluoride membranes (Merck). Membranes were blocked for 1 hour in 5% milk solution and incubated overnight with primary antibodies, followed by 1 hour with the appropriate horseradish peroxidase–conjugated secondary antibodies, as indicated in the legend of the figures. Immunodetections were performed using ECL reagent, and image acquisition was performed by using a chemiluminescent CCD imager (ImageQuant LAS 4000; GE Healthcare; or iBright 1500; Thermo Fisher Scientific). Densitometric analysis of the bands was performed with the ImageQuant TL or the iBright analysis software. The different antibodies used for western blot analysis are presented in the Supplementary Table.

### 2.11 ELISA

Platelet lysates were spiked with either 77.2 μM GTPyS or 771.6 μM GDP (30 minutes; 37 °C), diluted in lysis buffer, and analyzed via ELISA using 6 × 10^6^ platelets per well. In some experiments, endogenous active RAP1 was measured directly in lysed PRP before and after stimulation with ADP or TRAP14.

High-binding 96-well plates were coated with goat antihuman Fc (Fab')2 (Abcam) overnight at 4 °C, saturated for 1 hour at room temperature (sugar, 9%), and dried for 90 minutes at 41 °C. VHH-FcS solution (100 mL; 1.25 μg/mL) was added to the wells and incubated for 1 hour under agitation at 300 rpm. After washing, recombinant proteins, dilutions of platelet lysates (6 × 10^6^ platelets/well) loaded with GTPɣS or GDP or PRP before and after activation, were added to the wells and incubated for 1 hour under agitation at 300 rpm. In the case of PRP, incubation with lysis buffer was performed directly into the wells. Anti-Rap1 polyclonal or monoclonal antibodies (from Merck and Abcam, respectively) were tested for detection (1 hour incubation), followed by a 1 hour incubation with horseradish peroxidase–conjugated goat antirabbit (Jackson ImmunoResearch). After washing, the reaction was visualized by addition of chromogenic substrate TMB (Kementec) for 10 minutes. The reaction was stopped with 1 N H_2_SO_4_, and absorbance at 450 nm was measured using a microplate reader (Sunrise; Tecan). Soluble RhoA (CytobodX), Rap2A (Abnova), Rap2B (Abcam), and Rap1A (Abcam) were used to test ELISA specificity.

### 2.12 Statistical analysis

For data on recombinant proteins and platelet lysates, values reported are mean ± SEM of independent experiments. Unless indicated otherwise, *P* values were calculated with Prism 8 software (GraphPad) using a Student’s *t*-test or a 2-way analysis of variance (anova).

## 3 Validation

### 3.1 VHH selection

The first generation of VHHs recognizing the Rap1B were selected by sequential negative and positive selection from the synthetic phage display NaLi-H1 library using constitutively inactive Rap1B S17N and active Rap1B G12V, respectively (Supplemental Figures 1–3). The synthetic phage display NaLi-H1 library was first depleted of phages that bind inactive biotinylated MBP-Rap1B S17N attached to magnetic streptavidin beads as described previously [14]. After amplification, the phages that bind to active Rap1B were selected by affinity on immobilized MBP-Rap1B G12V. Unbound phages were removed by 10 to 20 washing steps with PBS + 0.1% Tween-20. Bound phages were eluted twice with 100 mM triethylamine for 10 minutes and neutralized with 1 M Tris, pH 7.4; *E coli* cells were infected with the eluted phages and plated on ampicillin containing agarose. One round of phage display is depicted in Supplementary Figure 2.

**Figure 1:**
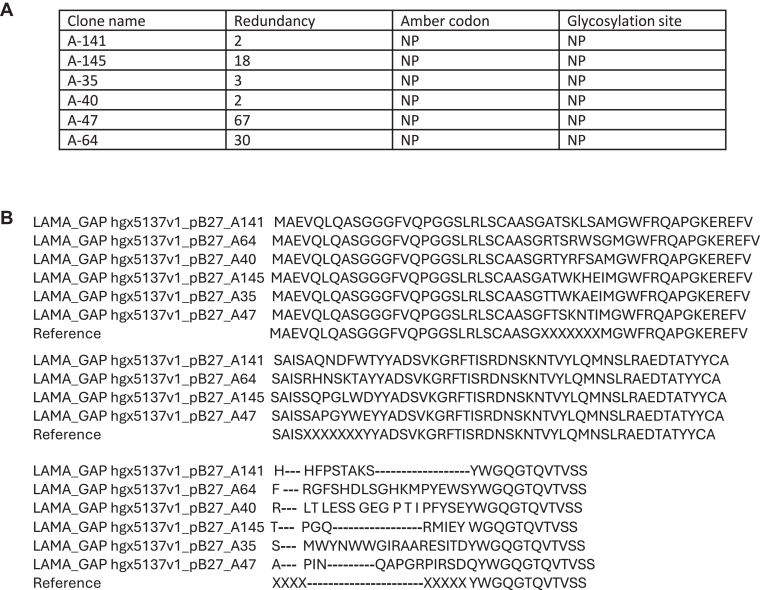
(A) Summary of the clone’s redundancy. (B) Alignment of the selected clones. Ref, a nanobody with CDR region replaced by X. NP, no stop codon or glycosylation sites were found in any listed clone.

**Figure 2:**
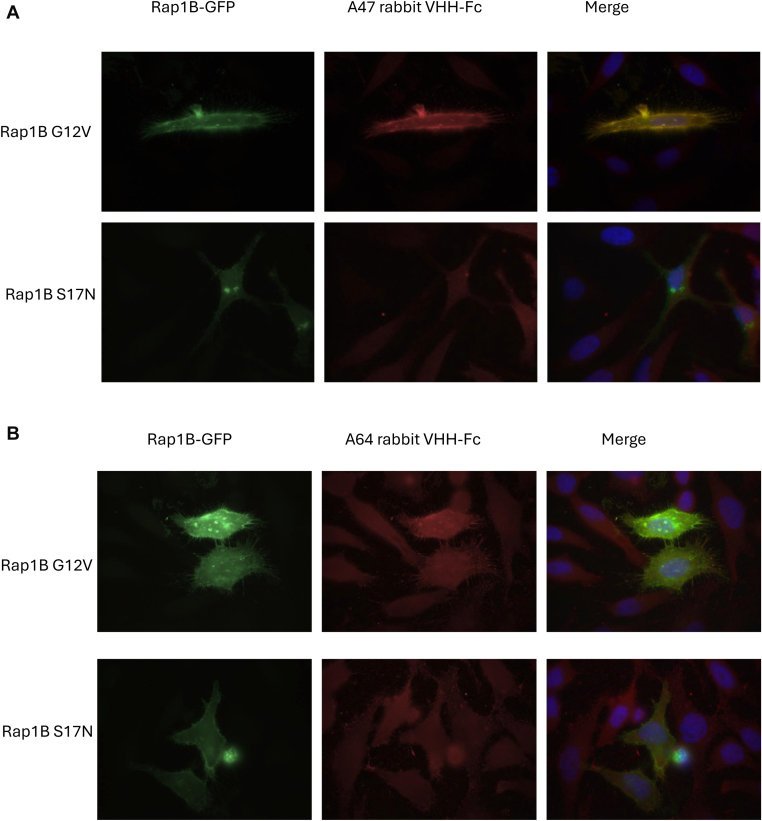
Recognition of Rap1B by nonpurified VHH-Fc A47 or VHH-Fc A64. HeLa cells were transfected with green fluorescence protein–tagged constitutively active (G12V) or inactive (S17N) Rap1B. Cells were fixed using paraformaldehyde, permeabilized with saponin, and stained with nonpurified rabbit VHH-Fc (pFuse supernatant), followed by Cy3-labeled antirabbit antibody, as described in the Methods section. Nuclei were stained using DAPI. (A) Staining results for VHH-Fc A47. (B) Staining results for VHH-Fc A64.

In order to validate the specificity of the selected VHHs toward the GTP-bound isoform and identify those that could potentially be used as intrabodies, interactions between VHHs and Rap1B G12V were tested via yeast 2-hybrid screening (Hybrigenics Services) [18,19]. The VHH sequences were cloned into the pP9 Yeast prey vector (derived from the original pGADGH plasmid). The sequence of active Rap1B (Rap1B G12V) was cloned into a pB27 plasmid as a C-terminal fusion to LexA (pB27 derived from the original pBTM116) [20]. Gap repair was used for assembling recombinant DNA in *Saccharomyces cerevisiae* (L40 strain). For screening, clones were tested using a mating approach with 532 YHGX13 (Y187 ade2-101::loxP-kanMX-loxP; matα) and L40ΔGal4 (mata) yeast strains as previously described [18]. The prey fragments of the positive clones were amplified by PCR and sequenced; 122 different VHHs clones were obtained, with redundancies ranging from 2 to 67. The sequences of the 2 clones with the highest redundancies (A47 and A64) (Figure 1) were subcloned in pFuse to produce VHH-Fc. VHH-Fc A47 colocalized with GFP–tagged active Rap1B G12V overexpressed in HeLa cells but not with inactive Rap1B S17N, suggesting that they are able to detect active Rap1B, whereas this was not the case for A64 (Figure 2).

**Figure 3:**
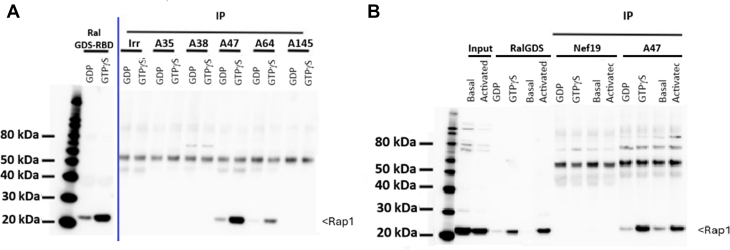
Immunoprecipitation of platelet lysates with VHH-Fc A47 and VHH-Fc A64. (A) Immunoprecipitation of platelet lysates loaded with GDP or GTPgS followed by western blot analysis. The results of VHH-Fc A47 and VHH-Fc A64 were compared with those obtained with an irrelevant VHH-Fc anti-GFP (irr) and ineffective anti-Rap1B VHH-Fc clones (A35, A38, and A145). All VHH-Fc samples were used at a concentration of 2 μg. The results were also compared with those obtained from a pull-down assay based on the interaction between Rap1 and RalGDS. Primary antibodies (rabbit polyclonal, diluted 1:500) and secondary antibodies (goat antirabbit, diluted 1:10,000) were obtained from Millipore. (B) Immunoprecipitation of platelet lysates under various conditions: basal (untreated), after GTPgS or GDP loading, and following activation with 50 μM thrombin receptor activator peptide (TRAP; activated). Western blot analysis was performed on the immunoprecipitates. Lanes 1 and 2 show platelet input before and after 50 μM TRAP activation. The negative controls correspond to immunoprecipitation using irrelevant VHH-Fc targeting Nef 19 protein (lanes 7-10). All VHH-Fc samples were used at a concentration of 2 μg. The results obtained with VHH-Fc silent A47 were compared with those of a pull-down assay based on the interaction between Rap1B and RalGDS (GDS-RBD) (lanes 3-6).

For potential use in cultured human cells and *in vivo* studies, Fc Silent VHH-Fc (FcS) fusion protein was produced and purified (Supplementary Figure 3). The ability of VHH-FcS A47 to capture active Rap1 was tested by immunoprecipitation on human platelet lysates incubated with GTPγS or GDP or on lysates of platelets stimulated with 50 μM TRAP6, followed by Rap1 immunodetection. Results were compared with those obtained with RalGDS pull-down experiment and with those obtained with VHH-Fc that do not target Rap1. VHH-FcS A47 efficiently captured Rap1 from GTPγS-loaded platelet lysates and lysates of TRAP6-stimulated platelets as effectively as RalGDS (Figure 3A, B).

### 3.2 VHH-Fc A47 optimization

**Figure 4:**
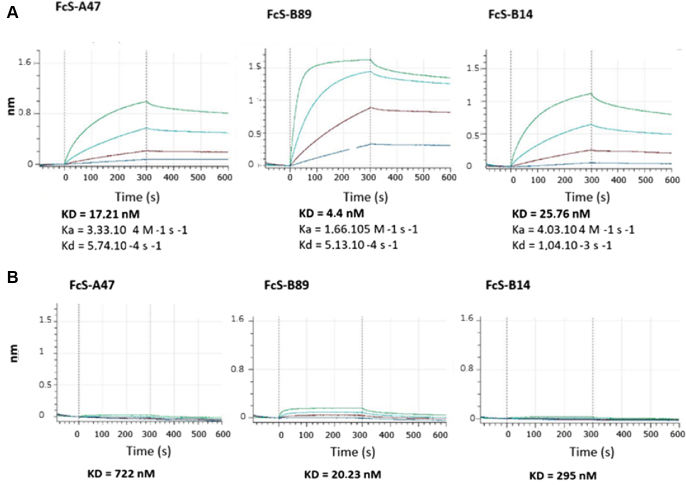
Using biolayer interferometry, we assessed the affinity binding curves of VHH-hFcS A47 and optimized VHH-hFcS B14 and VHH-hFcS B89. (A) The affinity of VHH-hFcS A47 and the variants VHH-hFcS B14 and VHH-hFcS B89 for recombinant GTPyS-bound Rap1B were assessed at 4 different concentrations for 600 seconds (300 seconds for the association step and 300 seconds for the dissociation phase). Affinity constants were calculated by subtracting the blank sample from the recorded data. (B) The affinity of VHH-hFcS A47 and the optimized VHH-hFcS B14 and VHH-hFcS B89 for recombinant GDP-bound Rap1B were measured at 4 different concentrations for 600 seconds (300 seconds for the association phase and 300 seconds for the dissociation phase). Affinity constants were calculated by subtracting the blank sample from the recorded data.

**Figure 5:**
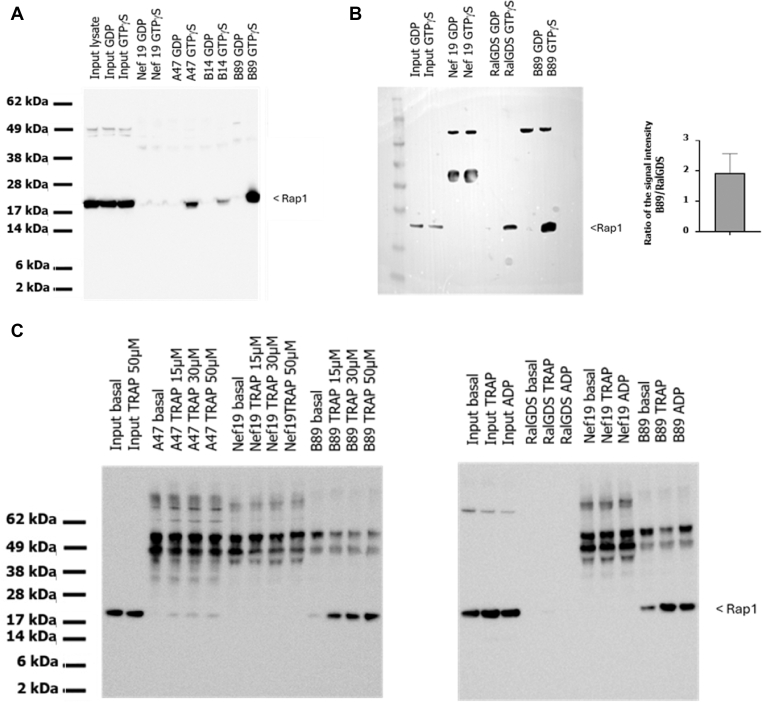
Immunoprecipitation of platelet lysates with VHH-Fc B89. (A) Immunoprecipitation of platelet lysates using VHH-Fc A47 and optimized VHH-Fc B14 and VHH-Fc B89, both before and after GTPgS loading. All VHH-Fc fusion protein was used at a concentration 2 μg. VHH-hFcS targeting Nef19 served as a negative control. (B) The results of VHH-Fc B89 were compared with those of RalGDS following GTPgS or GDP loading. The same protocol was applied for VHH-Fc B89 and RalGDS. The histogram displays the mean ± SD of the signal intensity ratio obtained with RalGDS over that of VHH-Fc B89 from 3 independent experiments. (C) The results from VHH-hFcS B89 were compared with those of VHH-hFcS A47 (left) and RalGDS (right) after platelet stimulation with thrombin receptor activator peptide (TRAP) or ADP at the indicated concentrations. We used VHH-hFcS targeting Nef19 as a negative control.

**Figure 6:**
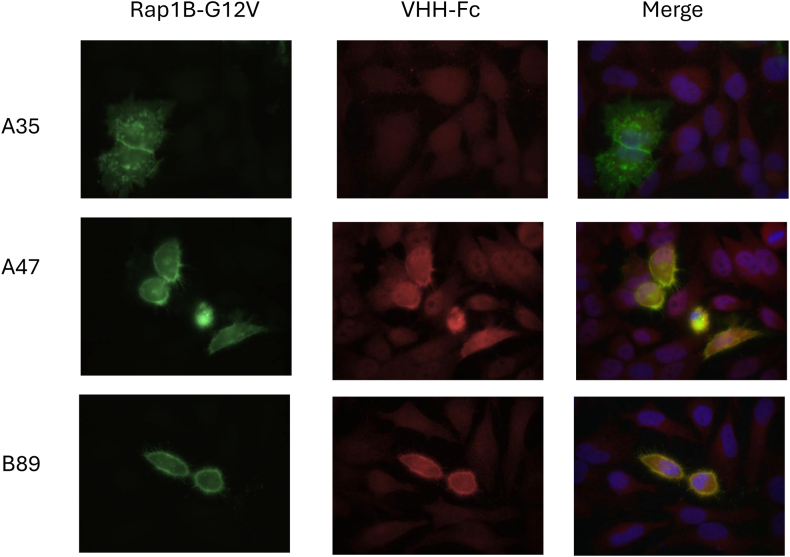
Recognition of Rap1B by nonpurified VHH-Fc A47 and VHH-Fc B89 in transfected HeLa cells. HeLa cells were transfected with green fluorescence protein–tagged constitutively active Rap1B G12V, fixed using paraformaldehyde, permeabilized with saponin, and stained with nonpurified rabbit VHH-Fc (pFuse supernatant), followed by Cy3-labeled antirabbit antibody. Nuclei were stained using DAPI. VHH-Fc A35 served as a negative control.

The sequence of the selected VHH (A47) was cloned into pP16 as C-terminal fusion to the Gal4 activation domain (Gal4AD-A47) under the control of the promoter Met. PCR-based mutagenic conditions were used to generate a mutagenesis library of 14.8 × 10^6^ independent clones in yeast. Rap1B G12V sequence was cloned as a LexA fusion, to identify strong binders compared with the initial VHH. Yeast 2-hybrid screening was carried out on a medium lacking tryptophan, leucine, and histidine (as the promoter Met controlling the expression of VHH can be leaky). Totally, 96 clones were processed across the 64 × 106 interactions tested. In parallel, screening was done on a medium lacking tryptophan, leucine, histidine, and methionine and supplemented with 5 mM 3-aminotriazol, where 142 clones were processed over 83 × 10^6^ interactions tested. Two VHH clones, B89 and B14 bearing mutations in CDR3 and FR2, respectively, were selected (Supplementary Figure 4). BLI was used to measure the binding constants of the original VHH-Fc A47 and the 2 optimized VHH-Fc (B89 and B14) captured on protein A biosensors and exposed to active GTP-bound Rap1B in solution (Figure 4). Among the 2 VHH-Fc, the affinity of B89 binding to Rap1B-GTP was improved (*K*_D_, 4.4 nM), with an association rate constant (*K*_a_) of 1.66 × 10^5^ M/s and a dissociation rate constant (*K*_d_) of 5.13 × 10^−4^/s (Figure 4A), while yielding a residual signal with Rap1B-GDP (Figure 4B). In line with its improved affinity, B89 provided better immunoprecipitation of active Rap1B from platelet lysates incubated with GTPγS (Figure 5A, B) and from platelets stimulated with TRAP6 (Figure 5C). Similar results were observed using mouse platelets, with excellent recognition of Rap1 after incubation of the platelet lysate with GTPγS and an absence of signal in case of GDP incubation, confirming the potential use of VHH-Fc B89 in mice (data not shown). VHH-Fc B89 colocalized with overexpressed active Rap1B G12V in HeLa cells (Figure 6), confirming its ability to selectively detect active RAP1B. Given the strong homology among Rap1B, Rap1A, and Rap2A, we assessed the reactivity of VHH-Fc B89 toward these different proteins. VHH-Fc B89 could efficiently precipitate Rap1A and to a lesser extent Rap2A (Supplementary Figure 5).

### 3.3 Development of an ELISA assay

**Figure 7:**
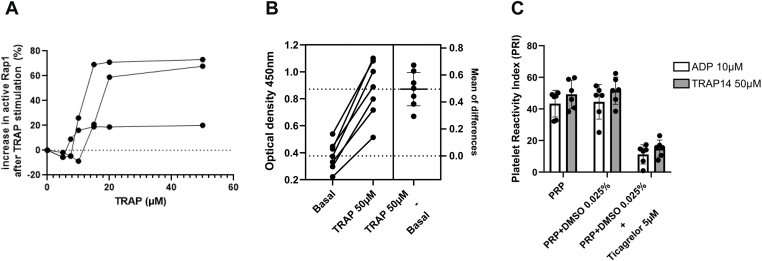
Rap1 activation following treatment with thrombin receptor activator peptide (TRAP) using the VHH-Fc B14–based ELISA assay. (A) TRAP14 induced a dose-dependent increase in active Rap1B levels in 3 different platelet-rich plasma (PRP) samples. The results are expressed as the percentage of active Rap1 calculated using the following formula: optical density (OD) after TRAP stimulation – OD in the basal state/OD after TRAP 50 μM stimulation. (B) OD values obtained from the PRP of 7 volunteers before and after TRAP (50 μM) stimulation. The mean differences are indicated on the right. (C) Changes in the platelet reactivity index in the presence of 5 μM ticagrelor. The X-axis displays the control conditions (PRP and PRP + dimethyl sulfoxide [DMSO] 0.025%) and the tested condition (PRP + DMSO 0.025% + ticagrelor 5 μM). The left Y-axis shows the platelet reactivity index expressed as a percentage and calculated as follows: (OD in stimulated conditions [ADP or TRAP] – OD in basal conditions) divided by OD in stimulated conditions (ADP or TRAP) × 100. The values are presented as the mean ± SEM from 6 different samples derived from volunteers. A significant decrease in the platelet reactivity index was observed in the presence of ticagrelor (*P* < .0001) under both stimulated conditions.

**Figure 8:**
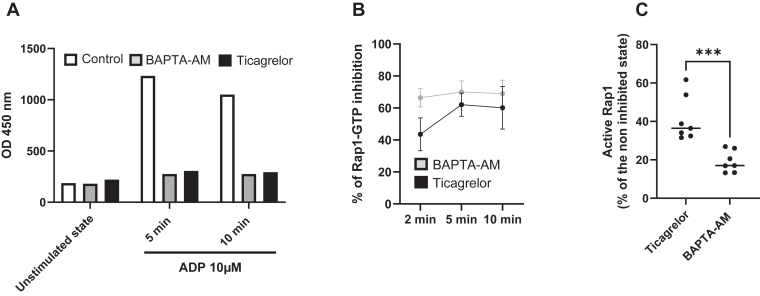
Activation of Rap1 Following treatment with BAPTA-AM or ticagrelor. (A) ADP (10 μM) induces an increase in Rap1-GTP levels in a platelet-rich plasma (PRP) sample (control). BAPTA-AM (200 μM) and ticagrelor (5 μM) were incubated with PRP for 30 minutes prior to stimulation with ADP for 5 or 10 minutes. Results are expressed as optical density (OD). During these stimulation periods, both inhibitors prevented Rap1-GTP formation. (B) The effects of BAPTA-AM (200 μM) and ticagrelor (5 μM) were evaluated over time at 2, 5, and 10 minutes following stimulation with thrombin receptor activator peptide (TRAP)-14 (50 μM). Results are expressed as the percentage of Rap1-GTP inhibition relative to the inhibitor-free condition at each time point. Two different PRP samples were analyzed. After 2 minutes of stimulation, ticagrelor was less efficient than BAPTA-AM. (C) Rap1-GTP levels were measured in PRP samples from 7 volunteers after 2 minutes of stimulation with TRAP-14 (50 μM), in the absence (control), or presence of ticagrelor (5 μM) or BAPTA-AM (200 μM). ∗∗∗Paired *t*-test, *P* < .001.

The 3 VHH-Fc clones—A47, B14, and B89—were used as capture antibodies to develop an ELISA assay to detect active Rap1 in human platelet lysates. Surprisingly, despite its relatively low affinity, VHH-Fc B14 demonstrated the highest efficiency in detecting GTP-bound Rap1 in platelet lysates when combined with the detection monoclonal antibody (Abcam), outperforming VHH-Fc A47 and B89 (Supplementary Figure 6). The amount of active Rap1 in PRP detected by ELISA increased with the concentration of TRAP6, reaching a maximum at 20 μM (Figure 7A). Using 50 μM of TRAP6, we observe a 2.3- ± 0.3-fold increase of signal (*P* < .0001, paired *t*-test) compared with that with nonstimulated PRP (Figure 7B). Ticagrelor is primarily used as an antiplatelet agent to prevent atherothrombotic events such as heart attacks and strokes. In this study, we examined its effects on Rap1 activation in PRP stimulated with ADP or TRAP. PRP from 6 different volunteers was enriched with ticagrelor (final concentration of 5 μM) or a control buffer containing 0.025% dimethyl sulfoxide and incubated for 1 hour at room temperature. Following incubation, PRP was stimulated with either 10 μM ADP or 50 μM TRAP14 for 10 minutes at room temperature before undergoing lysis and subsequent analysis via ELISA. The results showed that ticagrelor significantly inhibited Rap1 activation (*P* < .001), regardless of the stimulating condition. The activation of Rap1 is directly dependent on an increase in intracellular calcium levels. During platelet activation, Rap1 activation occurs very early and is subsequently reinforced by the secretion process, with ADP playing a central role. We compared the effects of BAPTA-AM, an intracellular calcium chelator that inhibits Rap1 activation at an early stage, with those of ticagrelor, a specific inhibitor of the ADP/P2Y12 pathway, which exhibits a delayed inhibitory effect. Both BAPTA-AM and ticagrelor effectively block Rap1 activation by ADP (10 μM) after 5 or 10 minutes of activation (Figure 8B). Notably, after 2 minutes of activation with TRAP-14 (50 μM), only BAPTA-AM strongly inhibits Rap1 activation, whereas ticagrelor requires a longer duration to achieve a similar level of inhibition (Figure 8B). We then assessed the percentage of active Rap1 after 2 minutes of activation with TRAP (50 μM) across 7 different PRP samples and confirmed that BAPTA-AM induces early inhibition of the active Rap1 form (18% ± 5.6% remaining active Rap1 in the presence of BAPTA-AM [200 μM] vs 40% ± 11.7% with ticagrelor [5 μM]; *P* < .001) (Figure 8C).The specificity of the ELISA was demonstrated by the absence of detection of recombinant RhoA, Rap2A, and Rap2B loaded either with GTPγS or GDP (not shown). Variation in 5 repeated measurements made on the same sample loaded with GTPγS under identical conditions gave a coefficient variation of 0.9%, whereas variation in 3 measurements made on the same sample loaded with GTPγS on 3 different days gave a coefficient variation of 6.5%.

## 4 Discussion

In this study, we are reporting the development and characterization of VHH against active Rap1. Most VHH available to date have been developed by animal immunization, but the development of synthetic in vitro platforms as used in this study is of great interest. Although we did not succeed in obtaining Rap1-GTP–specific VHH through the panning of an immune library obtained by immunizing 1 llama with recombinant Rap1B loaded with GTPγS (data not shown), we were able to select active Rap1-specific VHH through the panning of a synthetic library against both constitutively active (G12V) Rap1B and inactive (S17N) Rap1B. Our results demonstrate that Rap1B G12V and Rap1B S17N represent attractive tools [21] to select VHH against platelet active Rap1 although their constitutive activity may not be fully representative of the physiological signaling dynamics observed *in vivo*. This work confirms that synthetic VHHs can address the challenges of complex conformational targets that are poorly immunogenic, difficult for the immune system to recognize, or lose their conformation upon injection into tissues. Unlike traditional methods, synthetic libraries can demonstrate facilitated interactions even with hard-to-access epitopes.

From the panning against biotinylated Rap1 G12V, 2 rounds of selection were sufficient to select 6 nonredundant VHH. From this screening, potential VHH were isolated, with first A47 functioning as a superior VHH against active Rap1. The A47 VHH exhibited the highest redundancy and were able to specifically recognize Rap1B G12V transfected in HeLa cells but not Rap1B S17N with no sign of aggregation, indicating that this VHH is a conformation-sensitive antibody and suggesting that it could be useful for cellular detection of active Rap1. Analysis of platelet lysates confirmed the conformation selectivity of this VHH. The ability of VHH-Fc A47 to detect endogenous active form of Rap1 following platelet activation with TRAP6 demonstrates the possibility of using it as a substitute for RalGDS to measure the level of Rap1 activation in platelets. Two clones (B14 and B89) were selected following an optimization strategy. Kinetic analysis of VHH-Fc silent A47 and variants B14 and B89 were measured by BLI. A47 shows a good affinity for Rap1B loaded with GTPγS, with a *K*_D_ of 17.21 nM, whereas its affinity for Rap1-GDP was low as also reported for RalGDS [22,23]. Interestingly, the optimized VHH-Fc B89 shows an improved affinity 3 times higher than the VHH-Fc A47 (*K*_D_ = 4.4 nM), probably as good as or even higher than the one determined for RalGDS estimated close to 10 nM [22,23]. The improvement is likely supported by the presence of a mutation in the CDR3. The efficacy of VHH-Fc B89 in the immunoprecipitation assay surpassed that of A47. This is evident as an equal amount of antibodies yielded a more pronounced signal following both GTPγS loading and platelet activation. Furthermore, in our experimental conditions, B89 provides a signal strength twice as that obtained with RalGDS under comparable incubation conditions. The highly stable and resistance to extreme conditions, such as exposure to high temperatures or a wide range of pH levels as well as its large-scale and cost-effective production [24], could make it an ideal substitute reagent for RalGDS. This reagent could effectively detect both Rap1B and Rap1A in humans and mice.

Method to assess Rap1B activation are at best semiquantitative. The pull-down assay based on RalGDS relies on immunoblotting. In addition, this technique does not allow to evaluate the percentage of activated Rap1. Thus, the development of a quantitative method to assess Rap1 activation will be of great interest. In our ELISA assay, active GTP-bound Rap1 was captured by VHH-Fc, while inactive GDP-bound Rap1 was removed following washes. Captured active Rap1 was detected using a Rap1-specific antibody. It is interesting to note that the VHH-Fc A47 and VHH-Fc B89 exhibited inferior qualities compared with VHH-Fc B14 in recognizing the active form of Rap1 in ELISA assays. Indeed, VHH-Fc B89 generated a stronger background signal than VHH-Fc B14, while VHH-Fc A47 produced less intense signals. It is likely that the mutation in the vicinity of CDR2, which involves a change from an amino acid with an aromatic side chain to one with an aliphatic side chain, enhances the conformational recognition of active Rap1 by VHH-Fc B14. Its apparent lower affinity in this assay may not be a limitation in this setting, possibly because of an avidity effect that can be generated by the bivalent antibody subsequently used for Rap1 detection. In contrast, the VHH-Fc B14 is not suitable for immunoprecipitation and has consistently yielded poorer results than the B89 antibody, possibly because of the lower affinity in the absence of avidity. The B89 antibody is characterized by a mutation in the CDR3 domain, which is responsible for antigen recognition specificity. This alteration involves the substitution of a polar, hydroxylated serine with a nonpolar tryptophan that possesses an aromatic side chain, which not only induced a stronger binding affinity toward the GTP-bound form but also increased the affinity toward the GDP-bound form, possibly explaining the poor performances of this clone in ELISA.

Importantly, our results demonstrate that the B14-based assay is sensitive and specific, allowing the detection and quantification of the endogenous active Rap1 in the basal state or after platelet stimulation and the ability of Rap1 to bind GTPγS. It enables the assessment of the efficacy of ticagrelor, a potent antiplatelet agent, on Rap1 activation, thereby indicating its potential utility in evaluating platelet activation status in patients undergoing antiplatelet therapy. Additionally, the ELISA can detect early Rap1 activation, which is inhibited by 1,2-bis(o-aminophenoxy)ethane-N,N,N',N'-tetraacetic acid acetoxymethyl ester (BAPTA-AM), whereas ticagrelor takes a few more minutes to achieve its full effect. These data further confirm the specificity of the developed ELISA. Overall, our findings have allowed us to identify, for the first time, VHHs that can accurately measure the amount of active Rap1 inside platelets. This discovery positions these VHHs as promising tools for fundamental research. Additionally, we have also designed a reliable ELISA assay that should be easily implemented in clinical studies to assess platelet Rap1 activation in various medical settings.
