# Supplementary material for: Selection and optimization strategy for Rap1-targeting single-domain antibodies as platelet activation markers
Source: Res Pract Thromb Haemost. 2025 Dec 11;10(1):103294. doi: 10.1016/j.rpth.2025.103294 (PMC12860359; doi:10.1016/j.rpth.2025.103294)
Supplement: Supplementary Material [file mmc1.pdf]

Supplemental material

Supplementary Figure 1A

Sequence of the MBP-HA- Rap1B. The MBP sequence is highlighted in grey while the HA sequence is italicized and underlined. The substitution G12V and S17N are both bolded and underlined.

MBP-HA-Rap1B G12V. The start and the end of Rap1b are indicated by indices 1 and 184

MKIEEGKLVIWINGDKGYNGLAEVGKKFEKDTGIKVTVEHPDKLEEKFPQVAATGDGPDIIFWAHDRFGGYAQSG
LLAEITPDKAFQDKLYPFTWDAVRYNGKLIAYPIAVEALSLIYNKDLLPNPPKTWEEIPALDKELKAKGKSALMF
NLQEPYFTWPLIAADGGYAFKYENGHYDIKDVGVDNAGAKAGLTFLVDLIKNKHMNADTDYSIAEAAFNKGETAN
TINGPWAWSNIDTSKKVNYGVTVLPTFKGQPSKPFVGVLSAGINAASPNKELAKEFLENYLLTDEGLEAVNKDKP
LGAVALKSYEEELAKDPRIAATMENAQKGEIMPNIPQMSAFWYAVRTAVINAASGRQTVDEALKDAQTNSSSNNN
NNNNNNNLGIDTTENLYFQGAMG*YPYDVPDY*AAGRAM$_1$REYKLVVLGS**V**GVGKSALTVQFVQGIFVEKYDPTIED
SYRKQVEVDAQQCMLEILDTAGTEQFTAMRDLYMKNGQGFALVYSITAQSTFNDLQDLREQILRVKDTDDVPMIL
VGNKCDLEDERVVGKEQGQNLARQWNNCAFLESSAKSKINVNEIFYDLVRQINRKTPVPGKARKKSSAQLL$_{184}$

MBP-HA-Rap1B S17N

MKIEEGKLVIWINGDKGYNGLAEVGKKFEKDTGIKVTVEHPDKLEEKFPQVAATGDGPDIIFWAHDRFGGYAQSG
LLAEITPDKAFQDKLYPFTWDAVRYNGKLIAYPIAVEALSLIYNKDLLPNPPKTWEEIPALDKELKAKGKSALMF
NLQEPYFTWPLIAADGGYAFKYENGHYDIKDVGVDNAGAKAGLTFLVDLIKNKHMNADTDYSIAEAAFNKGETAN
TINGPWAWSNIDTSKKVNYGVTVLPTFKGQPSKPFVGVLSAGINAASPNKELAKEFLENYLLTDEGLEAVNKDKP
LGAVALKSYEEELAKDPRIAATMENAQKGEIMPNIPQMSAFWYAVRTAVINAASGRQTVDEALKDAQTNSSSNNN
NNNNNNNLGIDTTENLYFQGAMG*YPYDVPDYA*AGRAM$_1$REYKLVVLGSGGVGK**N**ALTVQFVQGIFVEKYDPTIED
SYRKQVEVDAQQCMLEILDTAGTEQFTAMRDLYMKNGQGFALVYSITAQSTFNDLQDLREQILRVKDTDDVPMIL
VGNKCDLEDERVVGKEQGQNLARQWNNCAFLESSAKSKINVNEIFYDLVRQINRKTPVPGKARKKSSAQLL$_{184}$

MBP-HA-Rap1B WT

MKIEEGKLVIWINGDKGYNGLAEVGKKFEKDTGIKVTVEHPDKLEEKFPQVAATGDGPDIIFWAHDRFGGYAQSG
LLAEITPDKAFQDKLYPFTWDAVRYNGKLIAYPIAVEALSLIYNKDLLPNPPKTWEEIPALDKELKAKGKSALMF
NLQEPYFTWPLIAADGGYAFKYENGHYDIKDVGVDNAGAKAGLTFLVDLIKNKHMNADTDYSIAEAAFNKGETAN
TINGPWAWSNIDTSKKVNYGVTVLPTFKGQPSKPFVGVLSAGINAASPNKELAKEFLENYLLTDEGLEAVNKDKP
LGAVALKSYEEELAKDPRIAATMENAQKGEIMPNIPQMSAFWYAVRTAVINAASGRQTVDEALKDAQTNSSSNNN
NNNNNNNLGIDTTENLYFQGAMG*YPYDVPDYA*AGRAM$_1$REYKLVVLGSGGVGKSALTVQFVQGIFVEKYDPTIED
SYRKQVEVDAQQCMLEILDTAGTEQFTAMRDLYMKNGQGFALVYSITAQSTFNDLQDLREQILRVKDTDDVPMIL
VGNKCDLEDERVVGKEQGQNLARQWNNCAFLESSAKSKINVNEIFYDLVRQINRKTPVPGKARKKSSAQLL$_{184}$

Supplementary figure 1B

Coomassie blue staining of MBP-HA-RAP1B (G12V and S17N) was performed at various stages of the purification process following SDS-PAGE gel migration. MW indicates molecular weight markers; NI refers to non-induced E.Coli; Ext denotes cell extracts ; R represents amylose resin; $E_{1\ to\ 4}$ correspond to successive elution. The expected molecular size of the MBP-HA-Rap1B is 70 KDa.

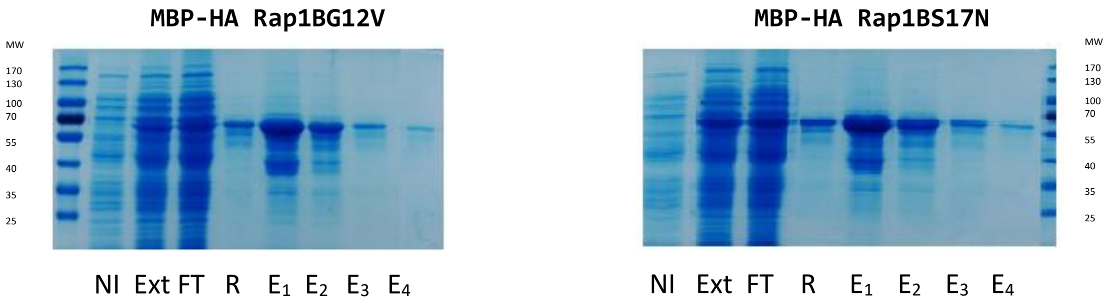

Supplementary figure 1C

2 micrograms of purified protein MBP-HA-RAP1B (WT, G12V and S17N) were subjected to pulldown experiments as described in the materials and methods section. The purified proteins were loaded with GDP or GTPγS and incubated with GST-RalGDS(RBD) which had been pre-coupled to glutathione-Sepharose beads. The beads were pelleted by centrifugation, and the bound Rap1B was eluted by adding SDS sample buffer. The samples were fractionated by SDS-PAGE, and Rap1B was detected by western blotting using anti-Rap 1 Rabbit polyclonal IgG Millipore (catalog number 07-916) diluted 1/500, followed by peroxidase- conjugated secondary antibody (thermos Fisher) corresponding to goat-anti-Rabbit diluted 1:10000. Inset: Western blot analysis of 100 ng of the three purified proteins.

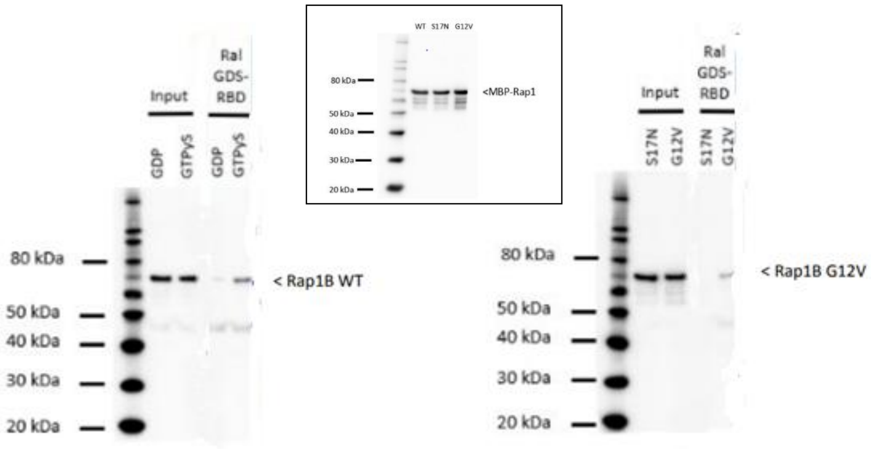

Supplementary figure 2

Schematic overview of phage-display strategy for enriching VHH that interact with active Rap1B G12V in an intracellular environment

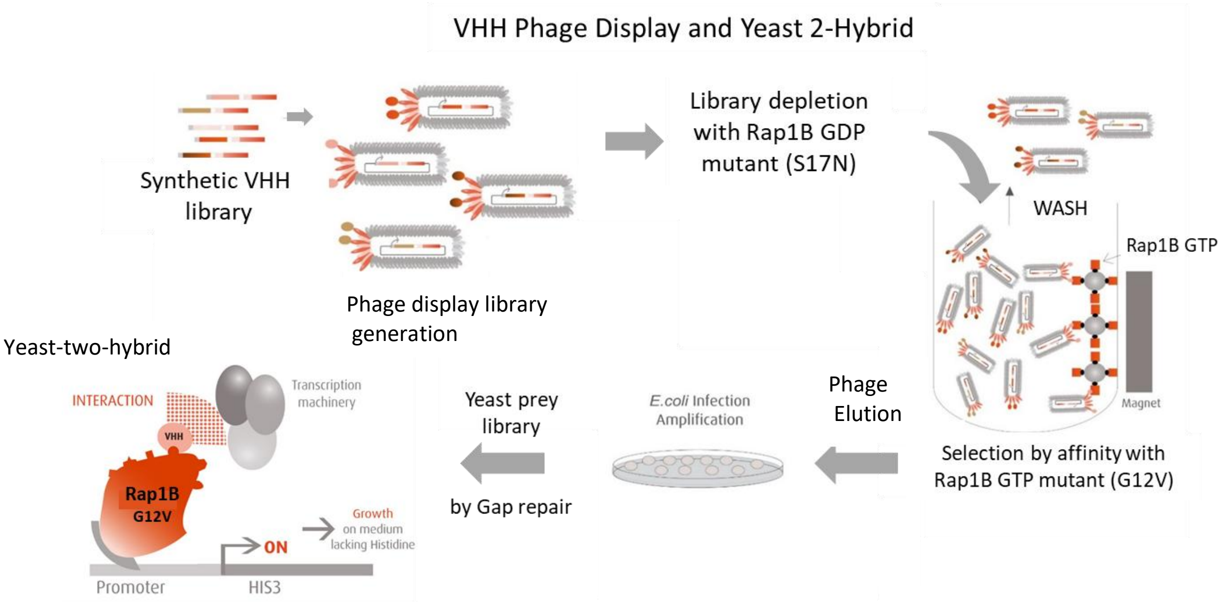

Supplementary figure 3

A total of 20 µl of each VHH-hFcS was loaded onto SDS-PAGE to evaluate homogeneity: irrelevant VHH-hFcS against Nef 19 (irr) (1.99mg/ml), A 47 (866 µg/ ml), A 64 (404 µg/ml)

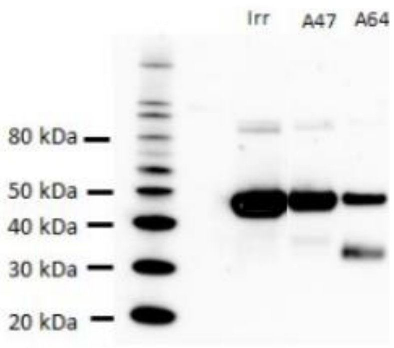

Supplementary figure 4

Sequences of the optimized nanobodies are presented with complementary determining regions (CDRs) highlighted in bold. Changes in framework region 2 (FR2) and CDR3 are underlined.

WT MAEVQLQASGGGFVQPGGSLRLSCAASGFT**SKNTIMG**WFRQAPGKERE**FVSAISSAPGYW**
B-89 MAEVQLQASGGGFVQPGGSLRLSCAASGFT**SKNTIMG**WFRQAPGKERE**FVSAISSAPGYW**
B-14 MAEVQLQASGGGFVQPGGSLRLSCAASGFT**SKNTIMG**WYRQAPGKERE**LVSAISSAPGYW**

WT EYYADSVKGRFTISRDNSKNTVYLQMNSLRAEDTATYYC**AAPINQAPGRPIRSDQ**YWGQG
B-89 EYYADSVKGRFTISRDNSKNTVYLQMNSLRAEDTATYYC**AAPINQAPGRPIRWDQ**YWGQG
B-14 EYYADSVKGRFTISRDNSKNTVYLQMNSLRAEDTATYYC**AAPINQAPGRPIRSDQ**YWGQG

WT TQVTVSS
B-89 TQVTVSS
B-14 TQVTVSS

Supplementary figure 5

Assessment of cross-reactivity of A47 and B89 with respect to Rap1A and Rap2A. Immunoprecipitation of platelet lysates was conducted using VHH-FcS A47 and VHH-FcS B89, both before and after loading with GDP or GTPγS. Each VHH-FcS was applied at a concentration of 2 µg. Specific antibodies targeting Rap1A (1:500) and Rap2A (1:500) were used.

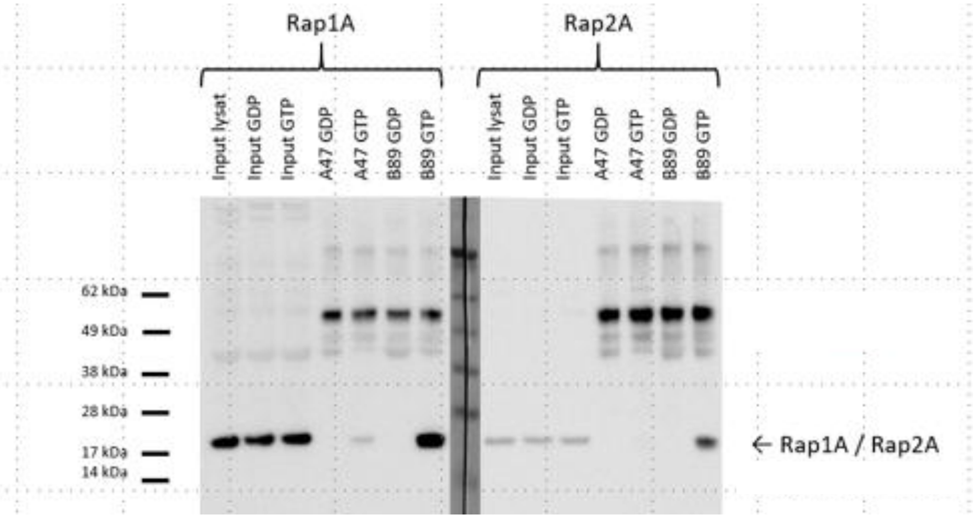

Supplementary figure 6

Performances of the ELISA assay for the VHH-FcS A47, B14 and B89. The ELISA was executed as described in the Material and Methods. Results from platelet lysates loaded with GTPgS or GDP are presented as mean ± standard deviation from two independent assays. Endogenous active Rap1 levels were measured in platelet lysates without any loading, while background levels were determined using buffer only. A / Results using polyclonal antibodies (Merck) as the reporter antibody B / Results using monoclonal antibodies (Abcam) as the reporter antibody. C / results obtained from PRP before and after activation with TRAP at 50µM using the B14 VHH-FcS/monoclonal antibody-based assay. Buffer was utilized to assess background levels.

A

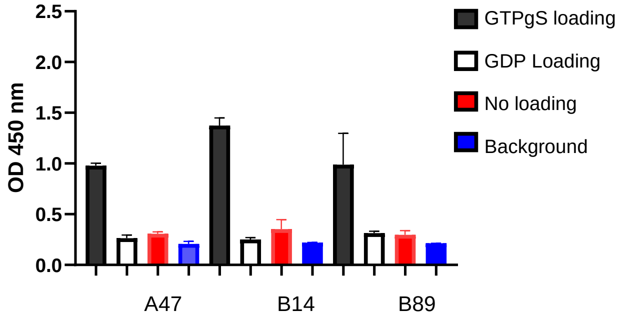

B

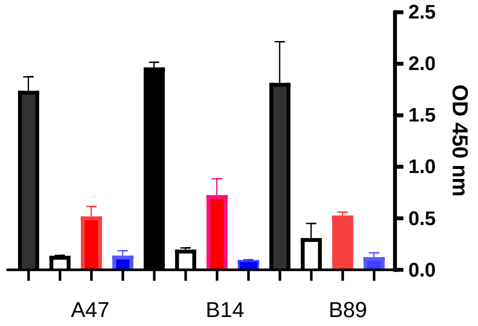

C

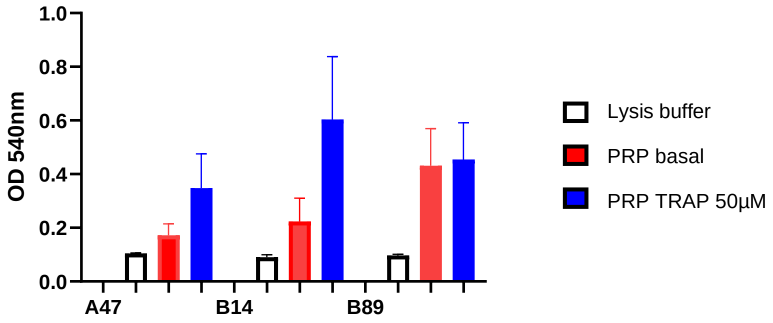

Supplementary table. Antibodies used for Western blot

primary antibody for Western blot	Merck, Cat. No. 07-916
primary antibody for Western blot	Abcam, EPR14815(B)
Secondary antibody for Western blot	Goat anti-Rabbit IgG (H+L) Invitrogen
Specific Rap1A antibody for Western blot	Invitrogen, MA5-17166
Specific Rap2A antibody for Western blot	Sigma, sab2107823
